# A case study on soil slope landslide failure and parameter analysis of influencing factors for safety factor based on strength reduction method and orthogonal experimental design

**DOI:** 10.1371/journal.pone.0300586

**Published:** 2024-05-15

**Authors:** Yongqing Zeng, Yinghuan Zhang, Weidong Hu, Meixin Chen, Qisheng Hu, Xiaohong Liu, Xinnian Zhu

**Affiliations:** 1 College of Civil Engineering and Architecture, Hunan Institute of Science and Technology, Yueyang, China; 2 Nanhu College, Hunan Institute of Science and Technology, Yueyang, China; 3 Department of Architecture and Civil Engineering, Hunan Urban Construction College, Xiangtan, China; Central South University of Forestry and Technology, CHINA

## Abstract

In civil engineering, stability analysis of slope is one of the main content of design. By using the finite element limit analysis software OptumG2, a landslide geological model is established to simulate the failure process of the landslide in Huadu District, Guangzhou City, China. The analysis focused on the deformation and failure characteristics, as well as the mechanical mechanism of landslide; the landslide mode of homogeneous soil is circular sliding. Additionally, investigating the influencing factors affecting slope stability is crucial in engineering implementation; in which the five influencing factors are considered as follow: slope height, slope gradient, soil cohesion, soil internal friction angle, and soil unit weight, respectively. A stability calculation model for a soil slope is established under 25 working conditions based on strength reduction method and orthogonal experimental design, in which the relationship between the safety factor and slope height, slope gradient, soil cohesion, soil internal friction angle, and soil unit weight is obtained. As the slope height increases from 5m to 45m, the safety factor of soil slope gradually decreases from 2.21 to 0.94; As the slope gradient increases from 20° to 60°, the safety factor of soil slope decreases approximately linearly from 1.80 to 0.95; As the cohesion of soil increases from 10kpa to 30kpa, the safety factor of soil slope increases approximately linearly from 1.04 to 1.60; As the internal friction angle of soil increases from 10° to 30°, the safety factor of soil slope increases approximately linearly from 1.00 to 1.81; As the unit weight of soil increases from 13kN/m^3^ to 21kN/m^3^, the safety factor of soil slope decreases approximately linearly from 1.50 to 1.21. The influencing factors affecting the safety factor of soil slope in descending order are slope height, slope angle, soil internal friction angle, soil cohesion, and soil unit weight. The research has reference significance for studying the stability and failure laws of soil slopes and conducting landslide control on soil slopes.

## 1. Introduction

In geotechnical engineering, the stability of natural soil slope is a critical problem [1]. In nature, the slope is usually in a state that can maintain its own stability; with the change of slope shape and the change of external force applied on the slope, the slope’s internal resistance and external disturbance force will also change. The slope is prone to instability and failure if the external disturbance force is greater or comparable with resistance forces [2, 3].

Slope stability is a significant problem that cannot be ignored in engineering construction management; slope stability is usually judged by the safety factor. Currently, the primary analysis methods include engineering analogy analysis, limit equilibrium analysis, numerical analysis, and uncertainty analysis [4]. Compared with the traditional limit equilibrium theoretical analysis method, numerical analysis can avoid spending a lot of time searching for the most dangerous sliding surface, thus improving the efficiency of engineering design [5–7]. Among the numerical analysis methods, the strength reduction method of finite element has attracted the attention of many researchers or designers because of its broad applicability, but it is difficult to quantify the stability coefficient; the limit analysis method requires pre-setting the most dangerous sliding surface; the finite element limit analysis method can take into account the advantages of these two methods, without the need to set the most dangerous sliding surface in advance. It can also speed up the operation efficiency, which is suitable for the preliminary design calculation of slope, tunnel, and other projects.

At present, both domestic and international scholars have conducted extensive research on the analysis of slope stability; which has been widely applied in various fields such as civil engineering, hydropower projects, and mining operations [8, 9]. The analysis methods for slope stability can be categorized into deterministic and non-deterministic approaches. Deterministic analysis methods include the limit equilibrium method and numerical methods. The former is typically represented by the Sweden Arc method and the simplified Bishop Method, while the latter includes approaches such as the finite element method and finite difference method. On the other hand, non-deterministic analysis methods include reliability analysis, fuzzy comprehensive judgment, grey system analysis, and artificial intelligence methods [10–14].

For theoretical formula derivation, the accurate calculation formulas are derived through the analytical method based on the safety factors of planar and circular failure surface slopes. The critical slip surface and minimum safety factor formulas for planar and circular failure surfaces are determined analytically [15, 16]. It is worth noting that the effective stress principle utilized in the finite element method can adequately reflect the loading effect of the acting force, the separate calculation method for water and earth pressure based on the effective stress concept encompasses all the water pressure present on slice boundaries as the loads acting on the slice; Consequently, the method can effectively represent the entirety of the acting force’s effects [17]. Slopes of granite residual soil are highly susceptible to deformation and failure when exposed to rainfall. Taking into account the initial moisture content, underground water level, and unsaturated characteristics of the soil, a comprehensive analysis based on the Green-Ampt model is conducted. Subsequently, an infiltration model that is applicable to various rainfall conditions is formulated; the accuracy and reliability of this model are confirmed [18]. To investigate the impact of spatial variability of soil strength on slope stability and test the effectiveness of the probabilistic method for evaluating slope stability, an improved and succinct Morgenstern-Price method was adopted to calculate the safety factor and the failure probability of the slope. The findings for a specific slope indicate that neither the safety factor nor the failure probability alone can provide a comprehensive assessment of slope safety, only the combination of two parameters is required for an effective evaluation of slope safety [19]. In order to gain a deeper understanding of soil slope failure, the virtual-bond general particle dynamics (VB-GPD) method has been developed for simulating slope stability; the results concluded that the VB-GPD method is highly effective in simulating soil slope stability and investigating soil slope failures [20]. The stability chart and Newmark model are commonly utilized for the evaluation of seismic stability in soil slopes. However, a novel method has been introduced to directly estimate static, pseudo-static safety factors, and critical accelerations of soil slopes, which effectively addresses the limitations associated with the stability chart and Newmark model [21]. The Lagrange element strength reduction method was employed to investigate slope stability and evaluate the underground mining of end-slope coal in a rock-stability analysis, which revealed distinct horizontal movement and deformation resulting from the underground mining disturbances [22]. A sensitivity analysis method for slope stability, based on the least squares support vector machine (LS-SVM), is proposed to evaluate the factors that influence slope stability, which were found to be consistent with the traditional Bishop analysis, thereby serving as a valuable reference for optimizing slope design [23]. A hybrid stacking ensemble approach is proposed to enhance the prediction of slope stability. The performance of the hybrid stacking ensemble approach was compared to that of the 11 individual optimized machine learning (OML) methods; a substantial improvement in slope stability prediction achieved by the hybrid stacking ensemble, surpassing the best performing individual OML method by 7% [24]. Soil slope failures are a common occurrence during the snow-melting season in regions with a cold climate, a new criterion called the Effective Precipitation (EP) index is introduced, and it shows that the new criterion is highly effective in predicting soil slope failures caused by snowmelt [25]. The study examines the impact of mobilized reinforcement tension on the stability of reinforced soil slopes at varying levels of soil-geosynthetic interaction; the Bishop Method is adapted to identify the critical slip surface and estimate the mobilized reinforcement tensile force [26].

For indoor model test and outdoor field test, with the implementation of the western mountainous railway construction in China, taking a railway high slope project as the basis and integrating field monitoring data, a research study was conducted that involved a combination of different parameters and a numerical simulation calculation model. The methodology facilitated the inversion analysis of mechanical properties of the rock and soil mass of railway high slope, as well as predicting its stability in the future stages [27]. For slopes composed of expansive soil, the main mode of failure is typically a shallow-layer slide, which can easily occur with repeated rainfall. A field testing program was conducted on five expansive soil slopes with varying inclinations; a hydro-mechanical analysis was conducted to estimate the safety factor of slopes with different patterns of fissures [28]. The accurate diagnosis of structural health for expansive soil slopes relies on rapid analysis of deformation monitoring data; a novel methodology is proposed for the purpose of health diagnosis; the study demonstrates the applicability of this innovative approach in identifying regions that display abnormal deformation in expansive soil slopes [29]. Numerous landslides often occur in soil slopes that overlay bedrock during excavation; a series of centrifuge model tests were conducted on these slopes, which featured different bedrock shapes. These slopes experienced a progressive failure process when subjected to excavation conditions; the failure mechanism of slopes that overlay bedrock during excavation involves a significant coupling of deformation localization and failure [30].

For numerical simulation, to study the mechanical behaviors of Soil-rock-mixture (SRM) slopes, a combination of Discontinuous deformation analysis (DDA) and Smoothed particle hydrodynamics (SPH) is utilized, which reveal that the deformation of SRM slopes is directly related to the roundness of rock blocks, while being inversely related to the sorting coefficient of the rock blocks [31]. Dynamic centrifuge model tests and associated analyses were conducted in order to develop a methodology for simulating slope failure during earthquakes and to evaluate the force exerted by a sliding soil mass on a structure, which reveal that the widely used Hertz’s equation and an empirical equation based on fluid mechanics are not capable of accurately estimating the impact force. However, the material point method (MPM) is a highly effective tool for simulating slope failure behavior and evaluating the impact force generated by a sliding soil mass [32]. A novel numerical approach, namely the material point method (MPM), has been utilized to analyze the accumulation of pore water pressure within landslide bodies during impacts against various types of structures. The study reveals that pore water pressures within landslides undergo significant tempo-spatial changes during dynamic impacts, highlighting the indispensability of a hydro-mechanical coupled approach for a comprehensive landslide-structure interaction (LSI) analysis [33]. A novel three-dimensional distance potential discrete element method has been developed to model the kinetic process of landslides, The effectiveness of this new approach has been verified by conducting experiments on well-known benchmarks, which involve a block impact simulation, a sliding/toppling test of a joint rock slope, and the response of blocks to induced acceleration in the foundation. The obtained results exhibit a commendable congruity with the analytical solutions, validating the capability of the newly proposed method to accurately depict the microscopic mechanical behaviors and macro-motion characteristics of individual blocks [34]. When rainfall infiltrates the soil, the soil near the surface can become saturated, resulting in the formation of a wetting band. This wetting band has the potential to trigger shallow landslides on the slope; a study was conducted on unsaturated soil slopes during rainfall, which revealed a significant correlation between the potential sliding surface and the evolution of the wetting band during slope failure at the moment of minimum safety factor [35]. Rainfall can greatly impact slope stability by changing the moisture content of the soil. In order to evaluate the stability of the Azhuoluo slope in Guizhou province, China, numerical calculations were conducted based on the innovative double-strength discounting method; the proposed double-strength discounting approach effectively captures the fundamental characteristics of slope instability [36]. The 67-meter high reinforced soil slope (RSS) was successfully completed in December 2006; the RSS structure experienced a catastrophic collapse in March 2015. The collapse occurred in a compound failure mode, where the failure plane passed both beneath and partially through the reinforced soil mass [37]. The seismic performance of earth slopes plays a crucial role in geotechnical earthquake engineering due to the presence of multiple sources of uncertainty. The investigations highlight the substantial influence of the spatial variability of soil properties on permanent slope displacements. Moreover, it is observed that the uncertainty associated with the variability in the temporal and frequency characteristics of excitations holds even greater importance compared to the effect of the spatial variability of soil properties [38]. By integrating the finite difference method with a random field model, the study explore the effects of spatial variability and randomness in soil strength parameters on the load-settlement behavior of a strip footing positioned on a geocell-reinforced soil slope; which evaluates the impacts of the footing setback distance, water table position, and flexural rigidity of the geocell mattress on the load-settlement behavior of the footing [39].

In this study, using the finite element limit analysis software OptumG2, taking the landslide in Huadu District, Guangzhou City, China as an example, a landslide geological model was established to simulate the dynamic failure process of the landslide from initial deformation to instability and movement cessation. The deformation and failure characteristics and mechanical mechanism of the landslide are analyzed. In addition, the analysis of influencing factors affecting slope stability is an important aspect of engineering implementation; there are many studies on factors affecting slope stability both domestically and internationally, but the main influencing factors for different slopes are not the same. In order to study the influence of soil slope parameters on landslide stability, this article considers the influence of five factors on slope stability, including slope height, slope gradient, soil cohesion, soil internal friction angle, and soil unit weight. Strength reduction method is used to calculate the stability of a homogeneous soil slope under different working conditions. The analyses were performed in OptumG2 software and orthogonal experimental design is chosen in this study; orthogonal experimental design is a multi-factor and multi-level experimental research method, which can efficiently identify influencing factors and save time. The safety factor under each influencing factor is obtained as a variation relationship of slope height, slope gradient, soil cohesion, soil internal friction angle and soil unit weight.

## 2. Overall analysis of Huadu District landslide

### 2.1. Descriptions of Huadu District landslide

The development of geological disasters in Huadu District is relatively serious, with all small-scale and medium-scale geological disasters. According to the survey, as of 2017, there have been 56 collapses and 28 landslides in Huadu District. Except for one medium-scale landslide, all other collapses and landslides are small-scale geological disasters [40]. Timian Town is located on the northern edge of Huadu District in Guangzhou, bordering Conghua District in the northeast and Qingyuan City in the northwest, with a total area of 91.2 km^2^, in which the mountainous area accounts for 83.5% of the total area of the town [41]. The landslide area is located at the foot of the monadnock in the mountainous landform area. A mountain landslide occurred due to continuous rainfall. The landslide area is tongue-shaped, and scarps are seen at the rear edge and side walls; at the front edge, there is a soil accumulation of the landslide, which is about 66,000m^3^ and directly reaches the back wall of the house. The landslide impacts the residential buildings, posing a threat to the safety of the lives and property of people at the foot of the slope, with an estimated threat to property of 5 million Chinese Yuan.

### 2.2. Basic features of the landslide

#### (1) Geological conditions and spatial morphological characteristics

**Fig 1:**
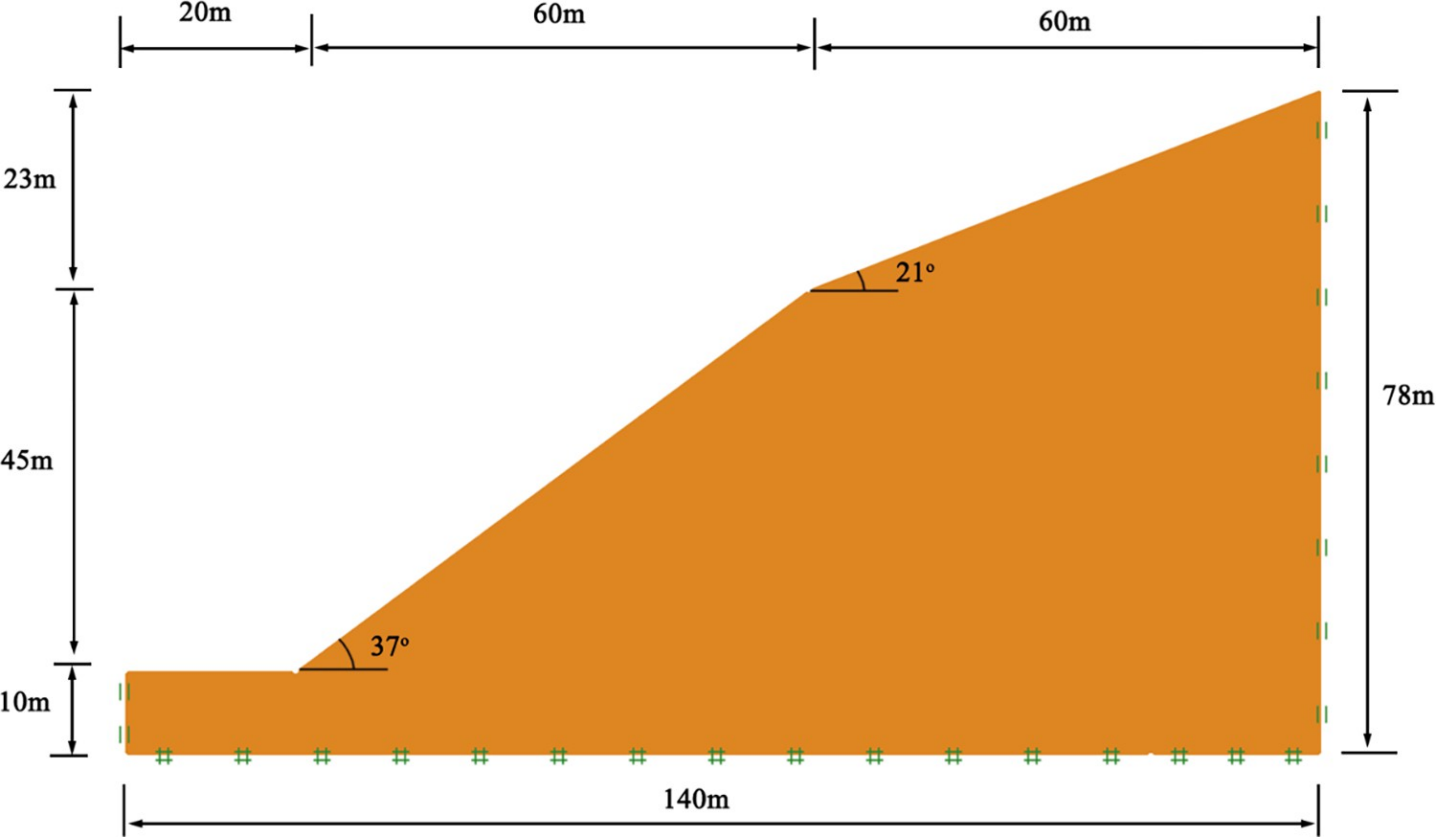
The longitudinal profile of the landslide at the Huadu District.

The landslide area is located at the foot of the residual hill slope in the mountainous landform area. The slope is composed of residual diluvial silty clay with a thick soil layer and low gravel content. The vegetation on the slope is eucalyptus, weeds, and shrubs. The highest point in the area is located at the top of the residual hill slope, with an elevation of about 105m. The lowest point is located in the village at the foot of the slope, with an elevation of about 37m and a relative height difference of 68m. The slope of the residual hill is gentle, with a 60m long, 23m high, and an angle of 21°. The foot of the slope is steep, with a 60m long, 45m high, and an angle of 37°. The longitudinal profile of the landslide at the Huadu District is shown in Fig 1.

#### (2) Landslide type and deformation failure mode

The slope surface is exposed and has poor stability. The landslide body is about 100m long, 55m wide and 12m thick. The plane of the landslide is tongue-shaped. The soil of the landslide body are exposed, with the front edge directly reaching the wall of the house and the rear edge having a steep height of about 12m. There are varying degrees of deformation and cracks in different parts of the sliding mass, among which obvious arc-shaped tensile cracks developed at the rear edge of the sliding mass, which are discontinuous. The prominent tensile cracks extend for about 20 meters, with steep ridges at the back edge and a downward displacement of 1 to 2 meters. The soil is loose in the middle and lower parts of the sliding mass, with dense secondary cracks and irregular development. Overall, it is distributed in a network and feather shape and has been eroded by rainwater. Cracks and scarps can be seen on both sides of the sliding mass, connected to the trailing edge scarp in a circular arc shape. The landslide has an area of approximately 5,500m^2^ and a total volume of approximately 66,000m^3^.

According to the classification of landslide volume, the landslide has a volume of about 66,000m^3^, less than 100,000m^3^; belonging to a small-scale landslide [42]. In terms of the classification of sliding mass types, the landslide is mainly composed of silty clay and belongs to the category of soil landslide. In accordance with the classification of mechanical properties, the landslide belongs to a composite with the upper part pushed and the lower part pulled. The deformation mode of the front edge of landslide belongs to the traction type; the high and steep excavation surface at the slope foot makes the slope body unstable due to impaired support. The deformation mode from the middle to the rear of landslide is the push type. After forming the landslide slip cliff, the back of the landslide loses support and forms tension cracks. The deformation mode of the landslide transitions from the traction type to the push type. The sliding control surface of landslide is an intra-layer dislocation zone, which generates vertical and longitudinal displacement along the sliding control surface and has strong destructive effects on the surface.

### 2.3. Numerical simulation and mechanism analysis of landslide failure process

#### (1) Introduction to OptumG2 numerical simulation software

OptumG2 is geotechnical analysis software that combines limit analysis and finite element analysis, which can perform two-dimensional finite element analysis. It has advantages in complex geological conditions, failure mode analysis of complex retaining structures, foundation-bearing capacity analysis, and reliability analysis [43, 44]. OptumG2 calculates the strict upper and lower limits for the physical quantities of concern; by utilizing the obtained upper and lower bound solutions, the exact solution and error range can be immediately estimated, and the accuracy of both can be improved by using more elements for calculation. Adaptive grid encryption can be used for analysis and provides a powerful method for high accuracy and low computational cost.

#### (2) Calculation principle of strength reduction method

The safety factor is an effective criterion to assess the slope slip stability and has been widely used in slope stability analyses; it is pointed out that the safety factor of a slope can be defined as the degree to which the shear strength of soil is reduced when the slope reaches the critical failure state [43, 45].

By gradually decreasing the shear strength index *c*, *φ*; dividing the value by the reduction coefficient F $F_{s}^{\prime }$ to obtain a new set of strength indicators *c*′, *φ*′; Performing finite element calculation and analysis, repeatedly calculating until the slope reaches the critical failure state, at this point, the ratio of the original strength index *c*, *φ* to the used strength index *c*_*f*_, *φ*_*f*_ of the soil is the safety factor *F*_*s*_ of the slope, as follows:

$$F_{s}=\frac{c}{c_{f}}=\frac{\tan\varphi }{\tan\varphi_{f}}$$ (1)

where *F*_*s*_ is the safety factor, *c* and *φ* are the original shear strength parameters, and *c*_*f*_ and *φ*_*f*_ are the shear strength parameters after strength reduction of the critical failure state.

By comparing the definition of safety factor between the Bishop method and the strength reduction method, it is believed that the safety factor of both methods has the same physical meaning, and the strength reduction method is consistent with the traditional method. The strength reduction method has the advantages of clear meaning and simple principles.

#### (3) Calculation of load case and parameter selection

A typical section parallel to the main sliding direction of the landslide is selected for calculation. The geometric dimensions of calculated model are described in Fig 1. The landslide area belongs to a site with a primary seismic intensity of VI, and the influence of seismic loads is not considered; there are no buildings on the surface of the landslide area, and the impact of surface loads is not considered. The investigation found that the soil at the foot of the slope is relatively moist, indicating that groundwater seepage in the landslide area has formed a certain dynamic water pressure, which has a certain impact on the stability of landslide. Moreover, on rainy days, the surface soil layer of landslide area is slippery and muddy, indicating that the soil layer of the landslide body is easily saturated due to rainwater immersion. If the water content of the soil is saturated, the saturation weight can be selected. Therefore, two conditions of self-weight combined with natural state and self-weight combined with heavy rainfall saturated state are selected for calculation.

According to on laboratory testing of site samples, the landslide sliding mass is mainly silty clay, with a natural unit weight of 14.6kN/m^3^ and a saturated unit weight of 19.7kN/m^3^. The strength parameters of soil under natural condition and saturated condition were test by consolidated undrained direct shear test. Under normal working condition (natural condition), the soil cohesion is 20.5kPa, the internal friction angle of soil is 28.9°, and under heavy rainfall condition (saturated condition), the soil cohesion is 14.2kPa, the internal friction angle of soil is 9.6°. The main calculation parameters of slope soil are shown in Table 1.

**Table 1:** The main calculation parameters of slope soil.

Name of soil layer	Natural unit weight(kN/m^3^)	Saturated unit weight(kN/m^3^)	Natural condition		Saturated condition	
			Cohesion (KPa)	Internal friction angle (°)	Cohesion (KPa)	Internal friction angle (°)
Silty clay	14.6	19.7	20.5	28.9	14.2	9.6

#### (4) Simulation results and discussion of slope in Huadu District

The criteria for landslide stability calculation and judgment refer to the national standard "Technical Code for Construction Slope Engineering" (GB50330-2013) [46], as shown in Tables 2 and 3. Due to the slope being a permanent slope with a height of 68m from the top to the foot of slope, the consequences will be very serious if a safety accident occurs. Therefore, the safety grade of slope engineering is first order; under normal conditions, the critical safety factor of slope *F*_*st*_ should be taken as 1.35.

**Table 2:** Classification of slope stability states.

Slope stability coefficient *F*_*s*_	*F*_*s*_<1.00	1.00≤*F*_*s*_<1.05	1.00≤*F*_*s*_<*F*_*s*t_	*F*_*s*_≥*F*_*s*t_
Slope stability states	Unstable	Understable	Basically stable	Stable

**Table 3:** Critical safety factor of slope *F*_st_.

Slope type		Safety grade of slope engineering		
		First order	Second order	Third order
Permanent slope	Normal condition	1.35	1.30	1.25
	Seismic condition	1.15	1.10	1.05
Temporary slope		1.25	1.20	1.15

Finite element limit analyses are performed using the strength reduction method to simulate the slope failure behavior and to evaluate the safety factor. The mohr-coulomb constitutive equation is used to analyze the stability of soil mass. The constitutive model can describe the stress and strain of soil mass, and the required parameters are easy to obtain [47]. This analysis considers boundary constraint conditions based on the selected typical cross-section; the left and right directions are semi-infinite soil space; therefore, the horizontal displacement on both sides of model is constrained. The bottom of the model believes that neither translation nor vertical movement can occur, so the horizontal and vertical displacements of the bottom of the model are constrained. The top of the model is not constrained to maintain the original free state of soil. The landslide is a shallow landslide. Through geological survey, it is found that the distribution of bedrock is deep and the sliding failure surface does not reach the bedrock level. Therefore, the influence of bedrock is not considered in the modeling process. When calculating the parameters of model, the long-term time condition is adopted, the element type is selected as the lower limit, the number of elements and the initial numbers of elements are 20000 and 2500, respectively, and the adaptive grid and four iterations are set.

**Fig 2:**
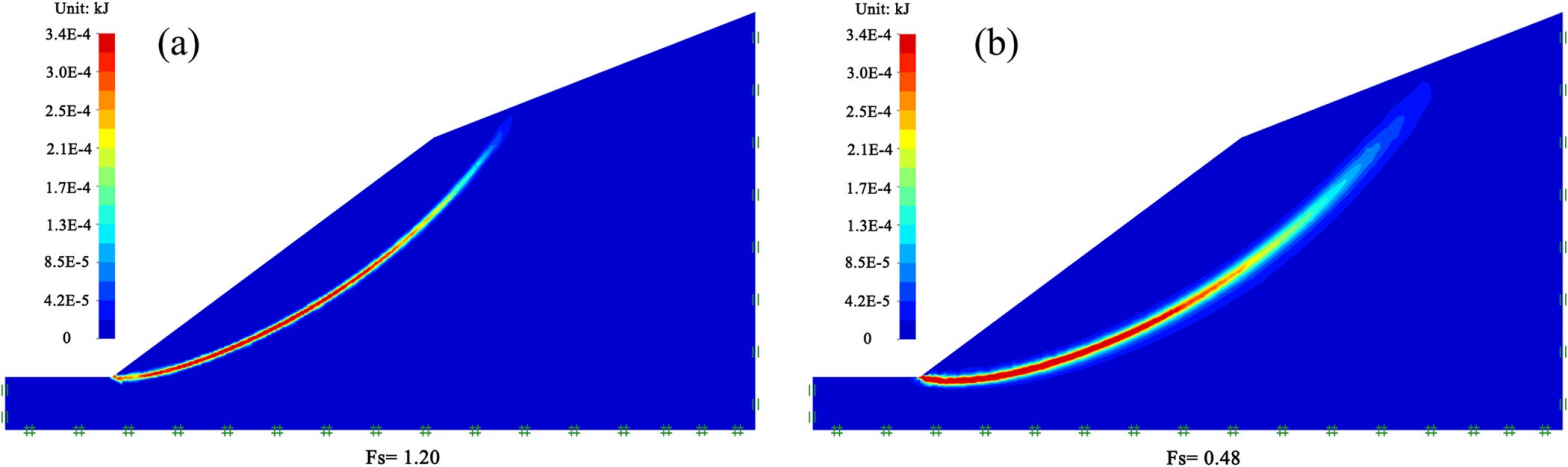
Potential sliding surface and safety factor of slopes. (a) natural condition; (b) saturated condition.

Using the OptumG2 numerical simulation software and the mechanical parameters described in Table 1, safety factors are calculated for natural and saturated conditions, respectively. The potential sliding surface and safety factor of the slope under natural conditions and saturated conditions are shown in Fig 2; a shear dissipation cloud map represents the possible sliding surface; the greater the shear dissipation energy, the greater the possibility of shear in this region, which is shown in redder in the figure. From Fig 2(A), it can be seen that when the slope is in natural conditions, the safety factor of the slope is 1.20, which is between 1.0 and 1.3. According to Tables 2 and 3, the slope is in a basically stable state; the potential sliding surface of the slope is a circular sliding surface that extends upwards from the bottom of slope, sliding out at the lower part of the second-level gentle slope. From Fig 2(B), it can be seen that when the slope is in the saturated condition, the safety factor of slope is 0.48, which is less than 1.0, it can be seen that the slope is in an unstable state; the potential sliding surface of the slope is also a circular sliding surface extending from the bottom of the slope; however, the sliding surface has a larger circular sliding radius, sliding out from the middle of the second-level gentle slope.

**Fig 3:**
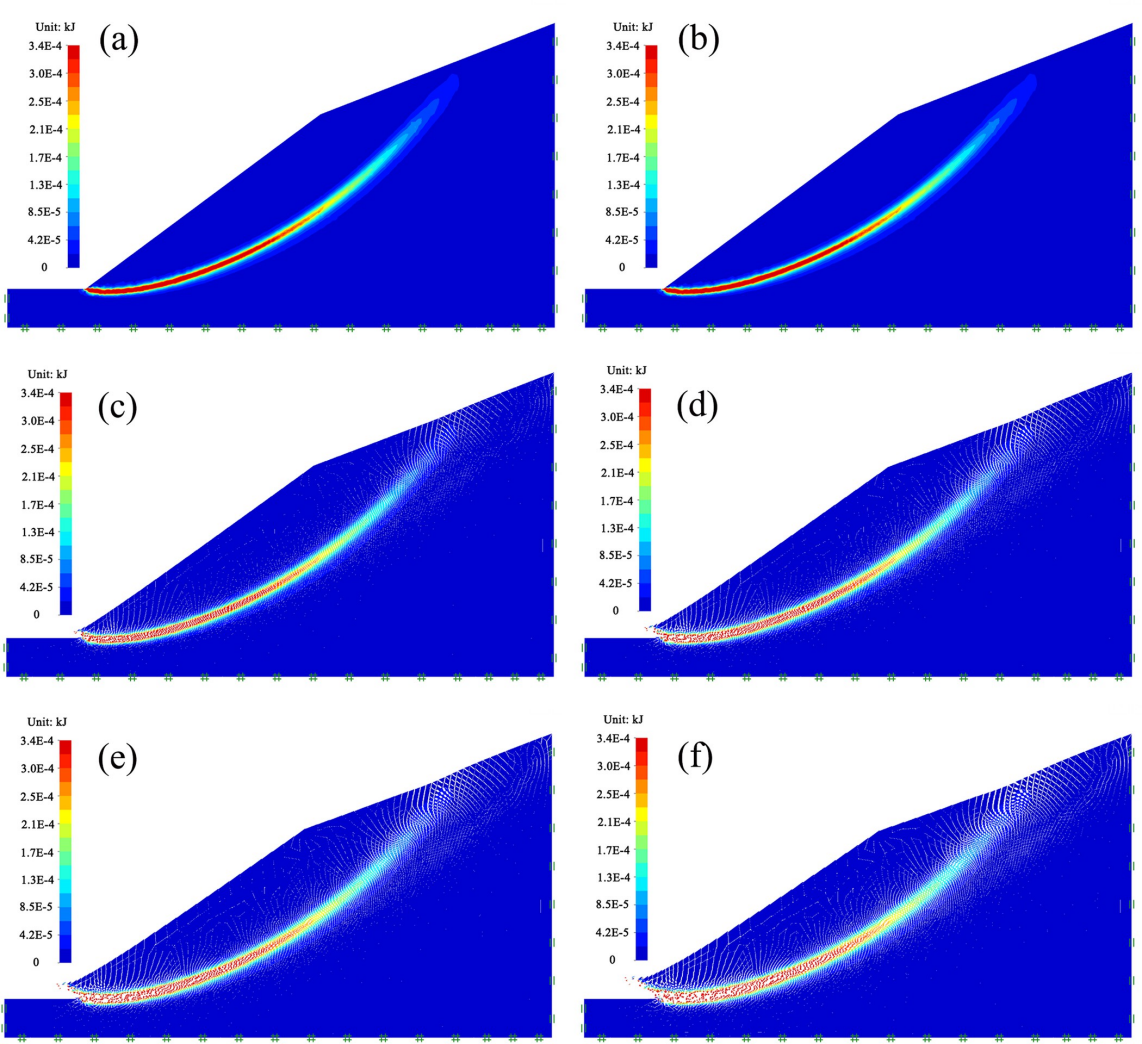
Simulation result of the slope landslide under saturated condition.

Since the safety factor of slope in the saturated condition is 0.48, which is less than 1.0, a landslide occurs on the soil slope. The simulation process of soil slope landslide is described in Fig 3. In the process of numerical simulation, when the penetration of plastic zone, the strain or displacement of the sliding surface changes sharply, the slope can be considered as failure, and the strength reduction factor at this time is the safety factor of slope. The instability of soil slope is a gradual process from local deformation accumulation and gradual expansion of regional instability to large deformation and overall failure. In the landslide of homogeneous soil, under the action of gravity, the slope slides downwards along the circular arc as a whole. The rupture surface is a circular sliding surface, and the displacement value at the slope foot is the largest.

It can be seen that the landslide law of the slope under saturated condition simulated by using OptumG2 is highly consistent with that of the site landslide failure characteristics; it is evident that OptumG2 can effectively simulate the landslide law of slope and provide a reliable approach for assessment of slope stability.

## 3. Parameter analysis of influencing factors for safety factor of slope

### 3.1. Orthogonal experimental design scheme and numerical simulation test results

**Fig 4:**
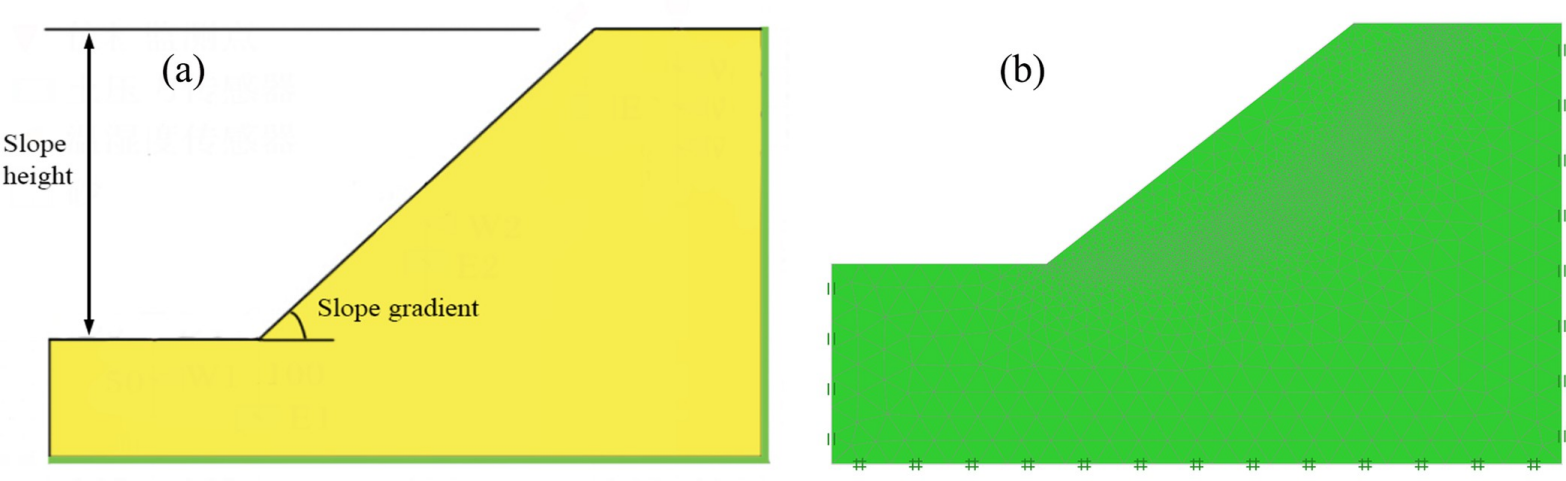
(a) A concept diagram of soil slope. (b) The finite element model of soil slope.

For the parameter analysis of influencing factors for the safety factor of slope, based on the finite element limit analysis software OptumG2, A concept diagram and the finite element model of soil slope are established, as shown in Fig 4. Based on engineering backgrounds such as highway roadbed, airport roadbed, railway roadbed, and mining slope, there is a large flat ground at the top of the slope.

Five factors affect the stability of slope: slope height, slope gradient, soil cohesion, soil internal friction angle, and soil unit weight. Each factor is selected at five levels based on engineering practice experience; a correlation of the investigated range of soil parameters to conventional values for slope design would be insightful; the values of soil parameters are determined according to the engineering geological manual on soil properties [48] and other relevant slope papers [49–51]. If a comprehensive combination test is conducted, 5^5^ = 3125 tests are required. Due to the limitations of time and money, it is unnecessary to perform 3125 tests.

By using the orthogonal table to arrange tests, the number of tests can be greatly reduced without affecting the comprehensive understanding of the influence of many factors on the performance index, the defects of blindness and the unrepresentativeness of test results in reducing factors or test times according to subjective experience can be avoided. At the same time, the orthogonal design method can be used to analyze the range and variance of different factors affecting the stability of slope and obtain the significant influencing factors.

Five horizontal values are used for each influencing factor to determine the impact of five factors on slope stability, including slope height, slope gradient, soil cohesion, soil internal friction angle, and soil unit weight, to obtain an orthogonal design table L_25_ (5^5^) [52, 53]. Stability calculations are performed on the 25 working conditions in Table 4 using the OptumG2 numerical simulation software, and the relationship between the safety factor and the changes in slope height, slope gradient, soil cohesion, soil internal friction angle, and soil unit weight under each factor is obtained, The numerical simulation test results of orthogonal experimental design for soil slope stability analysis are shown in Table 4, and the corresponding intuitive analysis table for orthogonal experiments is shown in Table 5. Here, the slope height is 5m, 15m, 25m, 35m and 45m, respectively; the slope gradient is 20°, 30°, 40°, 50° and 60°, respectively; the soil cohesion is 10kpa, 15kpa, 20kpa, 25kpa, and 30kpa, respectively; the soil internal friction angles is 10°, 15°, 20°, 25°, and 30°, respectively; the soil unit weight is 13kN/m^3^, 15kN/m^3^, 17kN/m^3^, 19kN/m^3^ and 21kN/m^3^, respectively. In addition, during the analysis, the young’s modulus E is 25MPa and the poisson’s ratio is 0.3.

**Table 4:** Results of numerical simulation test for soil slope stability analysis.

Test number	Slope height (m)	Slope gradient(°)	Soil cohesion (KPa)	Soil internal friction angle (°)	Soil unit weight (kN/m^3^)	Safety factor *F*_*s*_
1	5	20	10	10	13	2.00
2	5	30	15	15	15	2.11
3	5	40	20	20	17	2.27
4	5	50	25	25	19	2.33
5	5	60	30	30	21	2.33
6	15	20	15	20	19	1.61
7	15	30	20	25	21	1.48
8	15	40	25	30	13	2.25
9	15	50	30	10	15	1.18
10	15	60	10	15	17	0.63
11	25	20	20	30	15	2.30
12	25	30	25	10	17	1.08
13	25	40	30	15	19	1.03
14	25	50	10	20	21	0.53
15	25	60	15	25	13	0.87
16	35	20	25	15	21	1.31
17	35	30	30	20	13	1.68
18	35	40	10	25	15	0.86
19	35	50	15	30	17	0.96
20	35	60	20	10	19	0.31
21	45	20	30	25	17	1.80
22	45	30	10	30	19	1.20
23	45	40	15	10	21	0.41
24	45	50	20	15	13	0.69
25	45	60	25	20	15	0.61

**Table 5:** The intuitive analysis table for orthogonal experiments for soil slope stability analysis.

Test number	Slope height (m)	Slope gradient(°)	Soil cohesion (KPa)	Soil internal friction angle (°)	Soil unit weight (kN/m^3^)	Safety factor *F*_*s*_
1	1	1	1	1	1	2.00
2	1	2	2	2	2	2.11
3	1	3	3	3	3	2.27
4	1	4	4	4	4	2.33
5	1	5	5	5	5	2.33
6	2	1	2	3	4	1.61
7	2	2	3	4	5	1.48
8	2	3	4	5	1	2.25
9	2	4	5	1	2	1.18
10	2	5	1	2	3	0.63
11	3	1	3	5	2	2.30
12	3	2	4	1	3	1.08
13	3	3	5	2	4	1.03
14	3	4	1	3	5	0.53
15	3	5	2	4	1	0.87
16	4	1	4	2	5	1.31
17	4	2	5	3	1	1.68
18	4	3	1	4	2	0.86
19	4	4	2	5	3	0.96
20	4	5	3	1	4	0.31
21	5	1	5	4	3	1.80
22	5	2	1	5	4	1.20
23	5	3	2	1	5	0.41
24	5	4	3	2	1	0.69
25	5	5	4	3	2	0.61
Average of indicators ${\bar{K}}_{1}$	2.21	1.80	1.04	1.00	1.50	/
Average of indicators ${\bar{K}}_{2}$	1.43	1.51	1.19	1.15	1.41	/
Average of indicators ${\bar{K}}_{3}$	1.16	1.36	1.41	1.34	1.35	/
Average of indicators ${\bar{K}}_{4}$	1.02	1.14	1.52	1.47	1.30	/
Average of indicators ${\bar{K}}_{5}$	0.94	0.95	1.60	1.81	1.21	/
Extreme difference *R*	1.27	0.85	0.56	0.81	0.29	/

### 3.2. Numerical simulation results analysis and discussion

As the number of experiments is significantly reduced, it became crucial to choose an appropriate analysis method for the orthogonal experimental data. Range analysis is commonly conducted to reveal the main factors that impact the target index.

Range analysis aims to assess the impact of different factors on index. Two crucial parameters in range analysis are *K*_ji_ and *R*_j_. *K*_ji_ represents the sum of evaluation index at all levels (i, i = 1, 2, 3, 4, 5) for each factor (A, B, C, D, E). Meanwhile, ${\bar{K}}_{ji}$ refers to the average value of *K*_ji_. *R*_j_ is the range between the maximum and minimum values of ${\bar{K}}_{ji}$, and it is utilized to determine the significance of the factor [52, 53]. The calculation of R_j_ is as follows:

$$R_{j}=\max\{{\bar{K}}_{ij}\}-\min\{{\bar{K}}_{ij}\}$$ (2)

In range analysis, a larger value of R_j_ indicates a more substantial influence of the factor on the index; therefore, the corresponding factor is considered an important factor.

**Fig 5:**
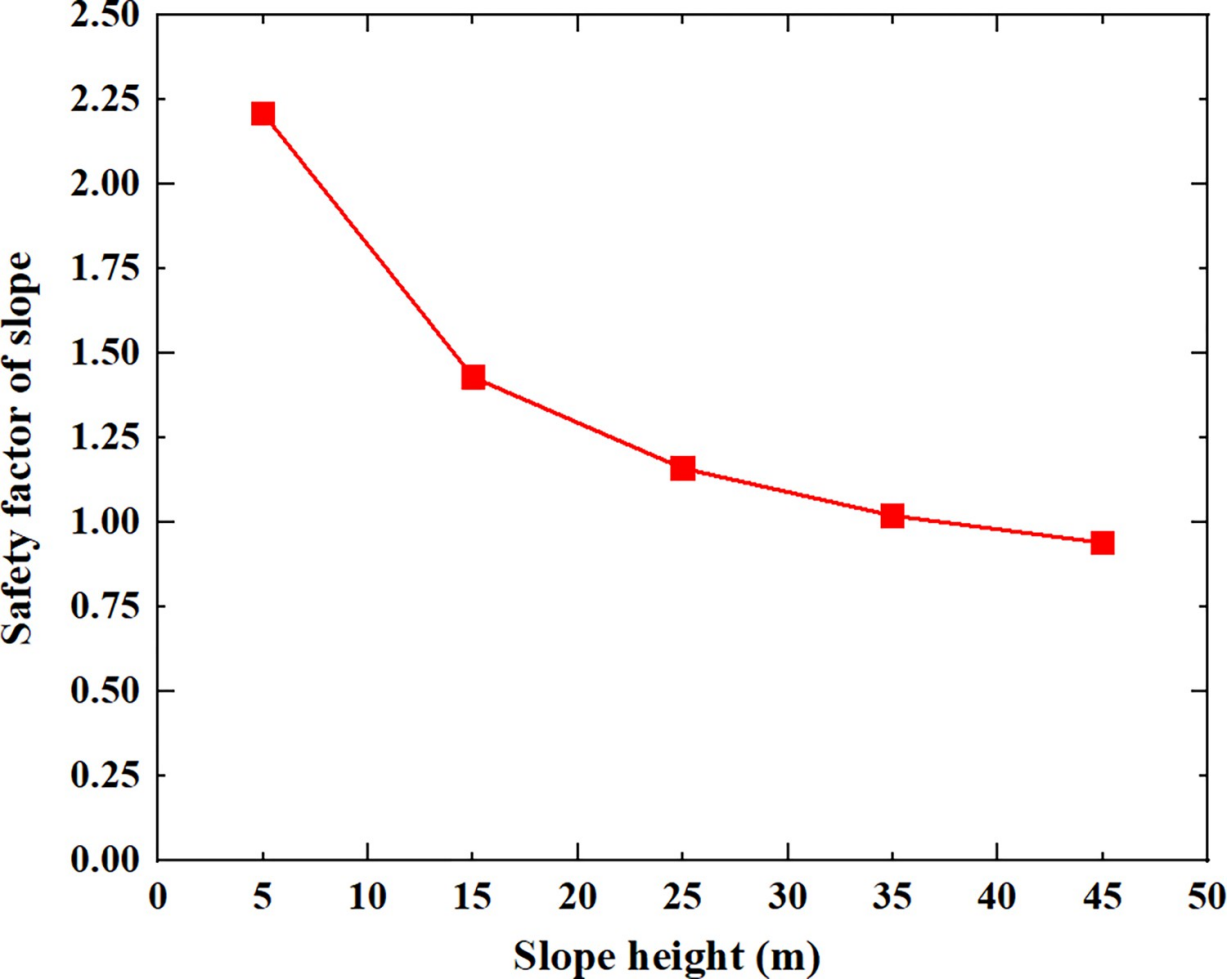
The relationship between the safety factor of soil slope and the slope height.

**Fig 6:**
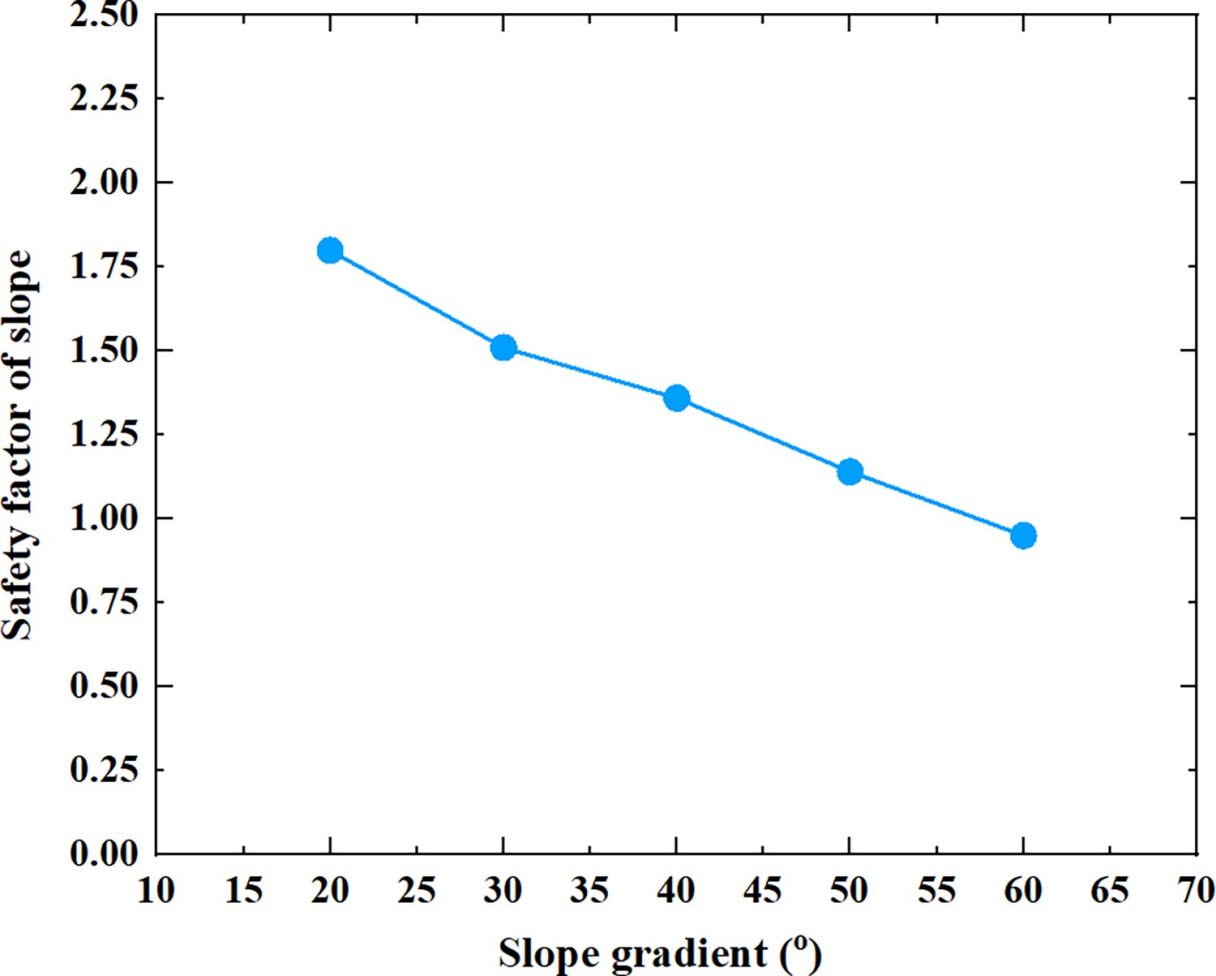
The relationship between the safety factor of soil slope and the slope gradient.

**Fig 7:**
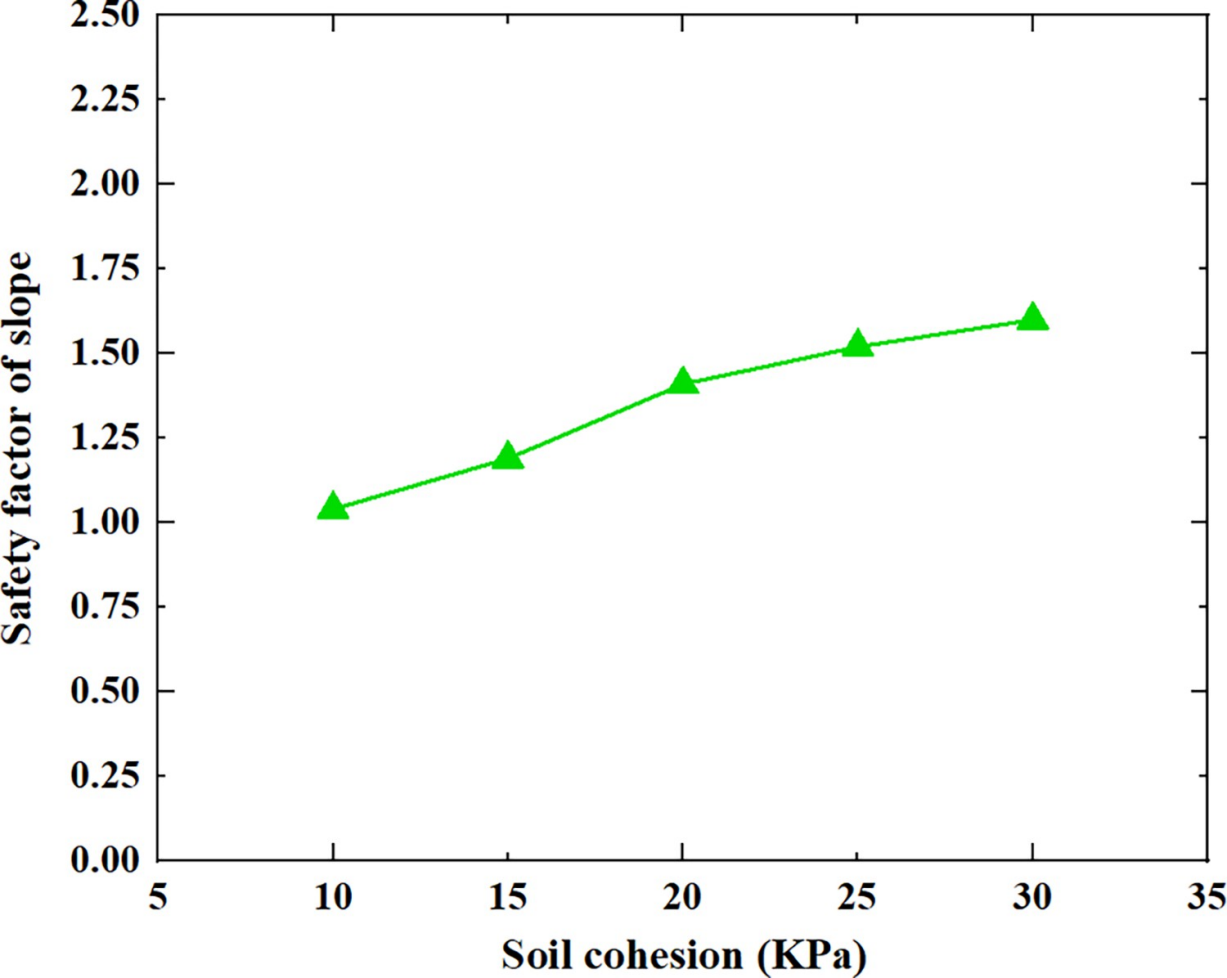
The relationship between the safety factor of soil slope and the soil cohesion.

**Fig 8:**
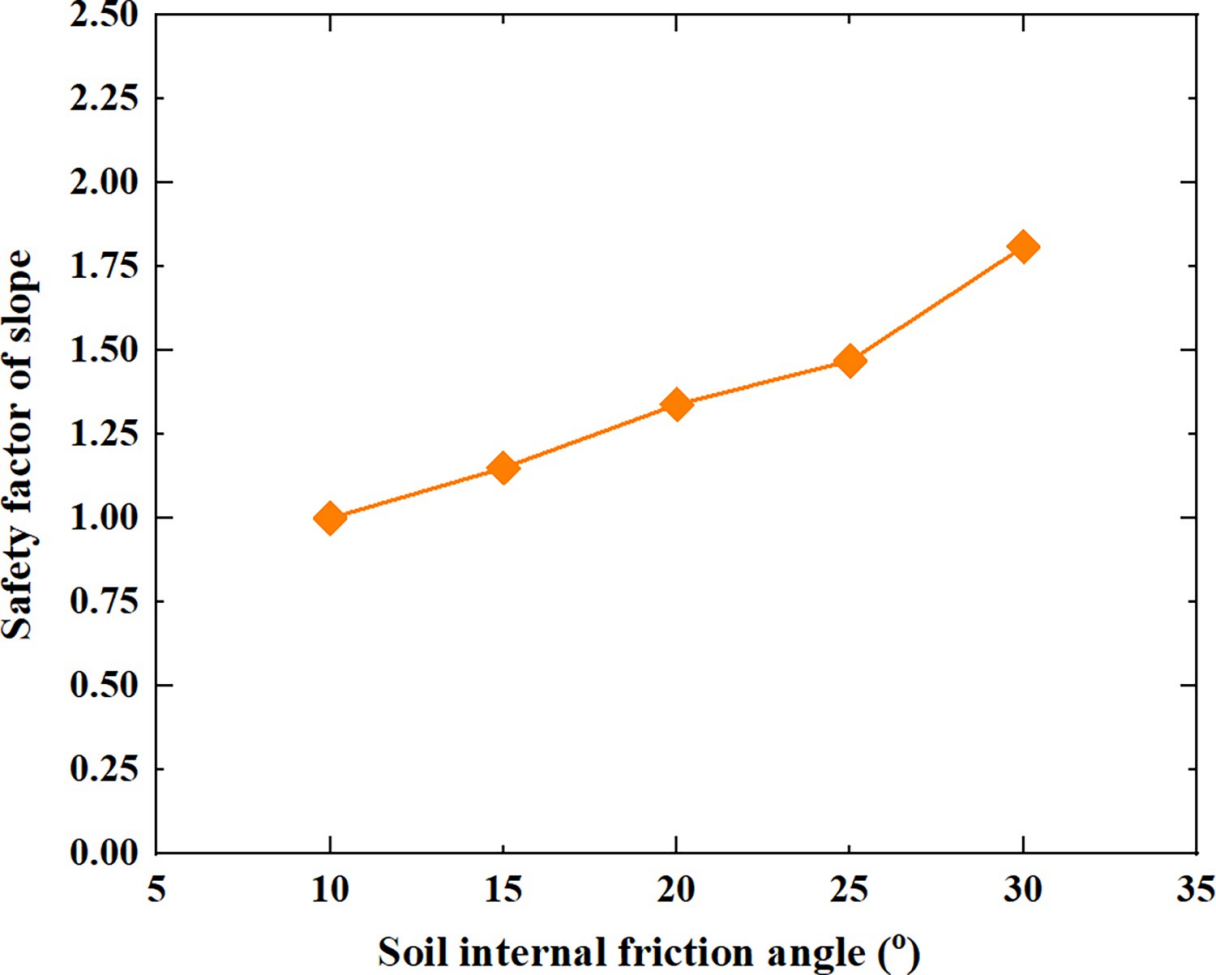
The relationship between the safety factor of soil slope and the soil internal friction angle.

**Fig 9:**
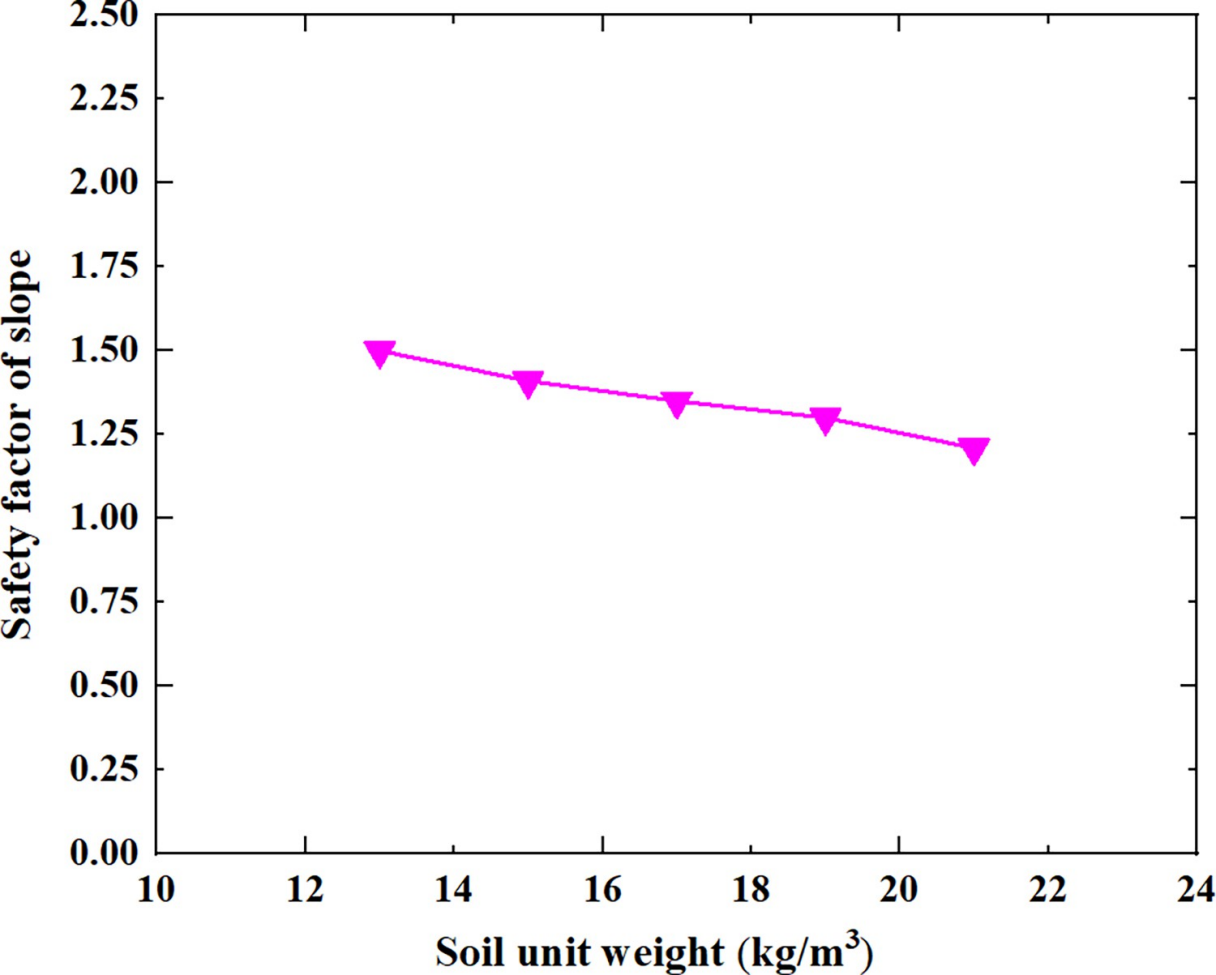
The relationship between the safety factor of soil slope and the soil unit weight.

Table 5 shows the relationship between the safety factor of soil slope and the slope height, slope gradient, soil cohesion, soil internal friction angle and soil unit weight shown in Figs 5–9.

From Table 5 and Figs 5–9, it can be seen that as the slope height increases from 5m to 45m, the safety factor of soil slope gradually decreases; when the slope height increases from 5m to 15m, the safety factor of soil slope decreases rapidly, from 2.21 to 1.43; when the slope height increases from 15m to 45m, the safety factor of soil slope decreases slowly and approximately linearly from 1.43 to 0.94; As the slope gradient increases from 20° to 60°, the safety factor of soil slope decreases approximately linearly from 1.80 to 0.95; As the cohesion of soil increases from 10kpa to 30kpa, the safety factor of soil slope increases approximately linearly from 1.04 to 1.60; As the internal friction angle of soil increases from 10° to 30°, the safety factor of soil slope increases approximately linearly from 1.00 to 1.81; As the unit weight of soil increases from 13kN/m^3^ to 21kN/m^3^, the safety factor of soil slope decreases approximately linearly from 1.50 to 1.21.

According to deformation observation data analysis of typical landslides, such as the Jiming Temple landslide, the deformation failure mechanism is summarized. Then, the mechanism is applied to slope stability analysis as the failure criterion by adopting the strength reduction method and gravity increase method; Xu [54] points out that the precision and reliability on the basis of the strength reduction method are better and can be applied to most slope stability analysis, when the cohesion of soil increases from 1kpa to 5kpa, the safety factor of soil slope increases from 0.85 to 1.14; when the internal friction angle of soil increases from 16° to 24°, the safety factor of soil slope increases approximately linearly from 0.85 to 1.25.

Connected with the engineering example, through the collection and analysis of related data, the site investigation and exploration of engineering geology, physico mechanical tests, adopting the analytic means of mathematic statistics, unbalanced-thrust method, and non-separation contact elastoplastic FEM, the influencing factor sensitivity of debris landslide has been analyzed and researched; Xu [55] found that the impact of decreasing slope gradient on the stability coefficient of gravel soil landslides is more sensitive than that of increasing slope gradient on the stability coefficient of landslides. As the slope gradient of landslide increases, the stability coefficient of gravel soil landslide decreases in an upward concave parabolic shape. As the cohesion of the sliding surface rock and soil increases, the gravel soil landslide stability coefficient increases linearly. As the internal friction angle of the sliding surface increases, the stability coefficient of the gravel soil landslide rises in a slightly upward concave parabolic shape. With the increased saturated area ratio of the sliding mass, the stability coefficient of the gravel soil landslide decreases approximately linearly. The change in elastic modulus of landslide rock and soil will not significantly impact the stability of gravel soil landslides. Aim at the sensitivity analysis of influencing factors of debris landslide, Xu [55] finds that through the sensitivity analysis of main factors that impact the stability of debris landslide, its main influenced factors are internal friction angle of sliding surface, topographic grade, saturation-area ratio of slope-mass and cohesion of sliding surface by descending order of sensitivity coefficients.

The extreme difference R in the orthogonal experimental analysis table reflects equilibrium, and the size of the extreme difference R reflects the influence of the factor change on the experimental index. The extreme difference R of slope height, slope gradient, soil cohesion, soil internal friction angle, and soil unit weight for the safety factor of soil slope are 1.27, 0.85, 0.56, 0.81, and 0.29, respectively, According to the order of range, the primary and secondary relationships of various factors are arranged from the largest to the smallest; it can be seen that the order of influence on the safety factor of soil slopes is: slope height, slope angle, soil internal friction angle, soil cohesion, and soil unit weight. The increase in slope height and slope gradient in engineering design and the increase in soil unit weight caused by rainfall will decrease slope safety and cause landslide damage. Increasing soil cohesion and soil internal friction angle in engineering reinforcement treatment can effectively improve the safety of soil slopes.

It should be pointed out that the order of influence on the safety factor of soil slopes may be different because the factor levels were determined in different ranges; the factor levels should be selected based on actual used values for slope design.

## 4. Conclusions

In this study, a landslide geological model is established to simulate the dynamic failure process of a landslide by using the finite element limit analysis software OptumG2; the deformation and failure characteristics, as well as the mechanical mechanism of the landslide, are analyzed. To study the effects of five influencing factors: slope height, slope gradient, soil cohesion, soil internal friction angle, and soil unit weight, on slope stability of slope soil, orthogonal experimental design is employed to calculate a homogeneous soil slope under 25 working conditions; the relationship between the safety factor and the slope height, slope gradient, soil cohesion, soil internal friction angle, and soil unit weight is obtained. The main conclusions are drawn as follows:

1. When the slope is in natural conditions, the safety factor of the slope is 1.20, the slope is in a basically stable state, and the potential sliding surface of slope is a circular sliding surface that extends upwards from the bottom of the slope, sliding out at the lower part of the second-level gentle slope. When the slope is in saturated conditions, the safety factor of slope is 0.48, the slope is in an unstable state, and the potential sliding surface of the slope is also a circular sliding surface extending from the bottom of the slope; however, the sliding surface has a larger circular sliding radius, sliding out from the middle of the second-level gentle slope.
2. As the slope height increases from 5m to 45m, the slope gradient increases from 20° to 60°, the unit weight of soil increases from 13kN/m^3^ to 21kN/m^3^, the safety factor of soil slope decreases from 2.21 to 0.94, from 1.80 to 0.95 and from 1.50 to 1.21, respectively; As the cohesion of soil increases from 10kpa to 30kpa and the internal friction angle of soil increases from 10° to 30°, the safety factor of soil slope increases from 1.04 to 1.60 and 1.00 to 1.81, respectively.
3. The order of influence on the safety factor of soil slopes arranged from largest to smallest can be observed as follows: slope height, slope angle, soil internal friction angle, soil cohesion, and soil unit weight. It is evident that an increase in slope height and slope gradient during engineering design, as well as an increase in soil unit weight due to rainfall, will result in a decrease in slope safety and potentially lead to landslide damage. On the other hand, enhancing soil cohesion and soil internal friction angle through engineering reinforcement methods can effectively improve the safety of soil slopes.

However, the result presented in this study is particularly for a simple and practical application in the landslide in Huadu District, Guangzhou City, China. Further research is necessary for more general applications. For example, in the present study, the spatial distribution of soil mass and the inhomogeneity of mechanical parameters are not considered. In the future studies, the effect of spatial distribution of soil mass and the inhomogeneity of mechanical parameters on behavior of the slope stability should be taken into account. Furthermore, the change of mechanical parameters of soil softening with water content should be further studied, and a relationship between slope stability coefficient and water content should be established. Moreover, the effects of the seepage on mechanical parameters of soil and slope stability coefficient should also be further studied.
